# ‘I did not think they could help me’: Autistic adults’ reasons for not seeking public healthcare when they last experienced suicidality

**DOI:** 10.1177/13623613251370789

**Published:** 2025-09-15

**Authors:** Tanya L Procyshyn, Rachel L Moseley, Sarah J Marsden, Carrie Allison, Tracey Parsons, Sarah Cassidy, Mirabel Pelton, Elizabeth Weir, Tanatswa Chikaura, Holly Hodges, David Mosse, Ian Hall, Lewis Owens, Jon Cheyette, David Crichton, Jacqui Rodgers, Simon Baron-Cohen

**Affiliations:** 1 University of Cambridge, UK; 2 Bournemouth University, UK; 3 Autism Action, UK; 4 University of Nottingham, UK; 5 SOAS University of London, UK; 6 East London NHS Foundation Trust, UK; 7 Ex GP research lead for East Dorset, UK; 8 Newcastle University, UK

**Keywords:** autism, mental health, public health, suicide

## Abstract

With autistic people at increased risk of dying by suicide, understanding barriers to help-seeking is crucial for suicide prevention efforts. Using an online survey designed in consultation with autistic people, we examined reasons why autistic adults living in the United Kingdom did not seek help from the National Health Service (NHS) when they last experienced suicidal thoughts or behaviours. Participants who disaffirmed help-seeking from the NHS (n = 754) were able to select from a prepopulated list of 20 reasons why and to enter their own. The three most commonly endorsed reasons were ‘I tried to cope and manage my feelings by myself’, ‘I did not think they could help me’ and ‘The waiting list is too long – no point’. Endorsement of reasons differed significantly with gender identity, age group and degree of lifetime suicidality. Four themes emerged from analysis of free-form responses: *NHS is ineffective*, *NHS as antagonistic, Fear and consequences* and *Barriers to access*. These findings highlight the need to foster more flexible healthcare systems capable of supporting autistic people, and that autistic people view as trustworthy and effective, to enable help-seeking behaviours with the potential to save lives.

**Lay abstract:**

Autistic people are more likely than non-autistic people to think about, attempt and die by suicide. For people in crisis, public healthcare services are, in theory, a source of help. In reality, many non-autistic people do not seek help from healthcare services. We wanted to understand why autistic people living in the United Kingdom may not seek help from the National Health Service (NHS) when suicidal and if these reasons differed by characteristics like age and gender. This study tried to answer these questions using responses from a survey co-designed with autistic people about various aspects of suicidal experiences. Participants were able to select from a list of 20 reasons and enter their own explanations (free-form responses) why they did not seek NHS support when suicidal. Our findings show that the most common reasons were that people tried to cope and manage by themselves; they did not think the NHS could help; and they thought the waiting list was too long. Reasons for not seeking help differed by age and gender, as well as lifetime history of suicidal thoughts and behaviour. For example, cisgender women and transgender/gender-divergent participants were more likely to say that previous bad experiences with the NHS prevented them from seeking help, and people with experience of suicide attempts were more likely to have been turned away by the NHS in the past. The free-form responses showed that many participants believed the NHS was ineffective, had previously had negative experiences with the NHS, worried about the consequences of help-seeking and experienced barriers that prevented help-seeking. This work highlights the crucial change and work required to make the NHS safe and accessible for autistic people so they can reach out for help when suicidal.

## Background

Autistic people experience poorer mental and physical health and live shorter lives than the general population (Catalá-López et al., 2022; Hand et al., 2020; Hwang et al., 2019; O’Nions et al., 2024). Suicide has emerged as a concerning contributor to this premature mortality, with large-scale studies reporting autistic people are 3–9 times more likely than non-autistic people to die by suicide (Hirvikoski et al., 2016; Kirby et al., 2019; Kõlves et al., 2021; Santomauro et al., 2024). The most recent meta-analysis of autism and suicidality, which pooled data from 80 studies, estimated that 1 in 3 autistic people have experienced suicidal ideation and nearly 1 in 4 have attempted suicide (Brown et al., 2024). These alarming statistics have led to a growing body of research on autism-adapted suicide prevention interventions (Huntjens et al., 2024; Rodgers et al., 2024) and motivated the inclusion of autistic people as a priority group in the Suicide Prevention Strategy for England (Department of Health and Social Care., 2023).

Suicide prevention – whether for autistic people or more broadly – is a complex endeavour due to the combination of individual, societal and systemic factors involved (Hawton & Pirkis, 2024). Nevertheless, primary healthcare services are seen to play a crucial role in suicide prevention because they serve as an accessible point of assessment and intervention (Lukaschek et al., 2024). General practitioners (GPs) commonly see patients experiencing mental health conditions like depression and anxiety, which are strongly associated with increased suicide risk (Moitra et al., 2021). Retrospective analyses of medical records show that the majority of people who die by suicide make contact with a primary healthcare provider in the 1-year period preceding their death (Cassidy et al., 2022; Stene-Larsen & Reneflot, 2019). People who die by suicide also show higher utilisation of healthcare services across various settings (primary care, hospital admissions, emergency rooms) than matched controls, with healthcare utilisation tending to escalate closer to their death (Ahmedani et al., 2019; Alothman et al., 2024; Chitty et al., 2023; John et al., 2020). These patterns suggest that healthcare providers have critical opportunities to support people at risk of suicide, particularly in the month prior to suicide attempts.

However, many people experiencing suicidality do not disclose this during healthcare-related interactions; indeed, many disclose to no one at all (Cassidy et al., 2022). A large cross-sectional survey found that only 26% of people with a history of suicidal ideation had ever disclosed to a healthcare professional (Husky et al., 2016). A psychological autopsy study of people who died by suicide in England found that 33% had communicated suicidal intentions before they died, with similar disclosure rates between individuals with and without evidence of autism (Cassidy et al., 2022). Despite availability of suicide screening instruments – including those adapted for autistic people (e.g. Cassidy, Bradley, et al., 2021; Hedley et al., 2025) – identification of suicide risk remains a challenge. Analysis of medical records of all individuals who died by suicide in Sweden in a single year found that, of those who contacted their GP during the last 30 days of their lives, only 6% had been identified as at risk (Öberg et al., 2024).

Understanding factors that prevent help-seeking is thus a crucial component of developing and implementing effective suicide prevention strategies. Prior research in the general population has identified the belief that treatment is not necessary, preference for self-management, fear of hospitalisation, stigma and structural factors (lack of time, financial constraints) as common barriers to help-seeking for suicidality (Han et al., 2018; Hom et al., 2015). Studies of specific groups offer more nuanced insights. Barriers to help-seeking most commonly reported by college students at high risk of suicide were the belief that treatment was not necessary, a lack of time and preference to self-manage, but not stigma (Czyz et al., 2013). A study of people aged 16–25 receiving mental health support found the most common reason for not disclosing suicidal ideation to a healthcare professional was concern about confidentiality (McGillivray et al., 2022). Among Australian men experiencing suicidal ideation but not receiving professional mental health support, the three most strongly endorsed barriers to help-seeking were preference to solve problems independently, dislike of talking about emotions and concerns related to effects on their family (Reily et al., 2024). Internalisation of masculine norms is associated with reduced help-seeking in transgender men and transmasculine individuals (Thomas et al., 2023), and the wider LGBTQIA+ community may avoid help-seeking for mental health due to previously encountered stigma and fear of discrimination (McNair & Bush, 2016).

With autistic people at increased risk of dying by suicide, there is urgent need to understand the specific barriers to help-seeking they encounter and whether these challenges vary based on factors like age and gender, as they do in the general population. Focusing on autistic residents of the United Kingdom, this study examined their reasons for not seeking support from the publicly funded National Health Service (NHS) when they last experienced suicidal thoughts or behaviours. Participants were able to select from a list of 20 reasons co-produced with autistic people and, to ensure no reasons were overlooked, add their own. By gaining a deeper understanding of what deters autistic individuals from help-seeking, these findings can contribute to the development of more accessible and effective health systems and provide valuable insights to inform targeted suicide prevention policy.

## Method

### Participants

Participants were drawn from a larger online survey on autistic people’s priorities for suicide prevention (see Supplementary Materials); the questions from which we derived the data described herein were not advertised as the focal point of the survey. The study was approved by the Psychology Research Ethics Committee of the University of Cambridge.

For the current analysis, the eligibility criteria were as follows: autistic (diagnosed or self-identifying), lifetime experience of suicidal thoughts and/or attempts, residing in the United Kingdom at the time of the study, responses deemed genuine by Qualtrics’ fraud detection measures and visual inspection, and completion of key survey questions related to help-seeking.

Of 1052 participants who met these criteria, 28.3% (n = 298) reported having sought NHS support when they last experienced suicidal thoughts or behaviours. This study purposively focuses on the 754 participants who did *not* seek NHS support. While the majority of these individuals sought no help at all (n = 570), some sought help from non-NHS sources (n = 184). As logistic regression confirmed these groups did not differ significantly in key demographic variables (age, ethnicity, highest educational attainment, current employment, diagnosed/self-identifying, gender; χ^2^(12) = 14.73, p = 0.256, Nagelkerke R^2^ = 0.03), they were combined as one group (n = 754) for quantitative analyses.

Qualitative analysis included the subset of participants (n = 140) who provided free-form responses why they did not seek NHS support. This sample was increased to 179 by including 34 participants who provided free-form responses as part of a pilot survey (see Supplementary Materials). Because the pilot survey included the same opportunity to provide a free-form response, but not the complete list of 20 reasons, these individuals could only be included in the qualitative analyses. As such, 18.9% of the qualitative sample was *not* included in quantitative analyses. Demographic information of participants included in the quantitative and qualitative analyses is presented in Table 1.

**Table 1.** Participant demographic information.

	Participants in quantitative analyses (n = 754)	Participants in qualitative analyses (n = 179)
Average age (SD, range)	35.63 (14.79, 16–89)	38.50 (14.40, 16–74)
Age groups		
% 25 and under	32.6	23.5
% 26 to 40	30.8	31.8
% 41 and above	36.6	44.7
Gender^a^		
% Cisgender men	25.3	24
% Cisgender women	53.6	54.7
% Transgender, gender-divergent or gender-questioning	21.1	21.2
Ethnicity		
% White	89.8	86
% Black	0.3	1.2
% Mixed or multiethnic	6	10.1
% Asian	1.5	0.6
% Other	1.4	1.5
% Undisclosed	0.09	0.6
Highest educational attainment		
% No formal qualifications above GCSEs, high-school diploma or equivalent	25.7	21.2
% AS Levels, A Levels, Access to Higher Education or equivalent	16.6	11.7
% Diplomas, certificate of higher education, degrees	34.4	34.6
% Postgraduate qualifications	21.9	31.8
% Prefer not to say/did not respond	1.5	0.6
Employment status		
% Any employment or student	67.1	67
% Caregiver or voluntary work	5	3.4
% Unemployed/unable to work	22.9	23.5
% Retired/did not disclose	4.9	6.1
Autistic status		
% Formally diagnosed	61.3	59.2
% Self-identifying^b^	38.7	40.8
Diagnosed co-occurring conditions		
% ADHD	18.7	21.8
% Anxiety	62.3	59.8
% Depression	62.2	63.7
% Eating disorder	15.5	17.3
% OCD	9.8	7.8
% Personality disorder	8.9	7.8
% PTSD or complex PTSD	19.8	24.6
% Sensory processing disorder	9.2	10.1
% Specific learning difficulty	15.9	17.3
Lifetime experience with suicidal thoughts/attempts		
% Brief passing thoughts only	11.3	12.8
% Suicide ideation without planning or attempts	24.3	22.9
% Suicide plans but no attempts	30.2	33
% At least one suicide attempt	34.2	31.3

a Over two questions, participants were asked their sex assigned at birth and current gender identity. For analysis purposes, these two questions were used to create a single item referred to hereafter as ‘gender’. The transgender, gender-divergent and gender-questioning group in the quantitative sample includes transgender men (17.6%), transgender women (3.8%), participants currently unsure of their gender (35.8%) and those who expressed a range of identities outside the binary (42.8%). In the qualitative sample, the transgender, gender-divergent and gender-questioning group includes transgender men (2.6%), transgender women (8%), participants unsure of their gender (44.7%) and those with varying non-binary identities (44.7%).

b The self-identifying autistic group includes individuals who were awaiting assessment at the time (62% of the overall sample and 57.5% of the qualitative sample).

### Procedures and measures

The survey began with questions about sociodemographic characteristics, including age, gender, sex assigned at birth, ethnicity, current employment and highest educational attainment. Subsequently, participants were asked about their lifetime experiences with suicidality (reported in Moseley et al., 2025) and ideas for suicide prevention (reported in Moseley, Procyshyn et al., In Preparation). The survey took approximately 20 min to complete (median and mode times of 21.6 and 11.6 min, respectively). At the end of the survey, participants were thanked and provided with mood mitigation and support resources (Townsend et al., 2020).

For the present study, key branching questions explored if and where participants sought support when they last experienced suicidal thoughts or behaviours. Participants who (1) sought no help at all or (2) sought help, but not from the NHS, proceeded to the central question of this analysis: reasons for not seeking NHS help. Participants were presented a list of 20 prepopulated reasons for not seeking NHS support (see Results: Table 2) and asked to select all that apply. These reasons were generated based on feedback from autistic people during the design phase, review of the broader literature on healthcare barriers faced by autistic people (Brede et al., 2022; Doherty et al., 2022) and feedback from a 2-week pilot period. Participants were also able to select ‘Other reason’ and enter a free-form response up to 200 characters in length.

**Table 2.** Proportion of respondents endorsing each reason for not seeking NHS support when they last experienced suicidality.

Reason for not seeking NHS support	n (of 754)	%
Tried to cope and manage my feelings	406	53.8%
Did not think they could help me	358	47.5%
Waiting list too long – no point	322	42.7%
Previous bad experiences seeking help for other things	273	36.2%
Previous bad experiences seeking help for suicidality	259	34.4%
Could not face trying to get GP appointment	257	34.1%
Did not know how to express my thoughts	248	32.9%
Did not know what help I needed	232	30.8%
Did not think I would be believed or taken seriously	224	29.7%
Thought of talking to anyone was too difficult	222	29.4%
Could not face attending GP appointment	211	28.0%
Worried about effect on others	200	26.5%
Afraid of being sectioned	188	24.9%
Worried about consequences	187	24.8%
Did not want medication/drugs	178	23.6%
Did not think it was necessary	176	23.3%
Did not know how or who to go to	125	16.6%
Previously turned away or referral rejected when suicidal	88	11.7%
Never thought of talking about it	41	5.4%
Did not want to be stopped	0	0.0%

#### Analysis

##### Quantitative analysis

Following data cleaning, we plotted the frequency with which participants endorsed each reason for not seeking NHS help. Subsequently, we performed two mixed analyses of variance (ANOVAs; alpha levels corrected to p < 0.025) to examine whether reasons for not seeking NHS help differed in relation to several between-subject variables of interest; in both, these reasons were modelled as a within-subject variable (‘Reasons’) with 20 levels, modelling participant endorsement (1) or negation (0) of each reason. First, Age and Gender were treated as categorical variables with three levels as per Table 1. In examining effects of these variables, we controlled for the following: Diagnostic status (formally diagnosed or self-identifying), Educational attainment and Current employment, categorised as per Table 1, and Ethnicity (collapsed to categorise participants as white or ethnic minority). Second, we controlled for Age and Gender in addition to these confounding variables to examine differences by participants’ degree of Lifetime Suicidality, a four-level variable. As sphericity was violated for the within-subject variable, Greenhouse–Geisser values are reported. Planned comparisons were performed where reasons for not seeking NHS support differed by Age, Gender and/or Lifetime Suicidality, including covariates and correcting alpha levels at an false discovery rate (FDR) of 0.05; where significant group differences were detected in relation to specific reasons for not seeking help, we report planned contrasts between cisgender men (reference category) against other gender groups, between those in the oldest age group (reference category) against other age groups, and between those with lifetime experience of suicide attempts (reference category) against other groups.

##### Qualitative analysis

To conduct thematic analysis, free-form responses were reviewed by two researchers (T.L.P. and R.L.M.) to establish comprehensive understanding of the content. An initial set of codes was collaboratively developed through discussions, which involved both researchers independently coding a subset of responses followed by meetings to compare, refine and consolidate the coding approaches. The entire dataset was then coded using the agreed-upon framework. Throughout this phase, the researchers revisited and revised the codes to capture the nuances of the data. The codes were then organised into broader categories to facilitate the identification of overarching themes. The final themes were established through further discussion and iterative refinement. To ensure rigour, the themes were reviewed in the context of the original data. Any disagreements or ambiguities were resolved through consensus. T.L.P. and R.L.M. maintained reflexive awareness of their positionality to the data throughout the analysis. The entire research group, including neurodivergent and neurotypical members, were privy to this analytic process (in addition to other analyses within this article) and read and ratified the interpretation, thus minimising particular influence of any one author.

Binary logistic regression indicated that older respondents were more likely to provide free-form responses (non-significant effects of other demographic factors; see Supplementary Table 1 for full details).

##### Community engagement

Our research team includes individuals who identify as neurodivergent, have lived or living experience of suicidality and/or suicide bereavement, and live or work closely with autistic people, including providing support for suicidal thoughts and behaviours; consequently, there is also collective experience within the group of seeking and/or supporting others to seek NHS help. Alongside our knowledge of the academic literature and practice landscape within the United Kingdom, these experiences have shaped our research questions and informed our data interpretation with diverse perspectives. During development, the survey was reviewed by an advisory panel of autistic people and their family members and revised accordingly.

## Results

### Endorsement of reasons

The full list of prepopulated reasons for not seeking NHS support and percentage of respondents endorsing each reason are presented in Table 2. The three most commonly endorsed reasons were ‘I tried to cope and manage my feelings by myself’, ‘I did not think they could help me’ and ‘The waiting list is too long – no point’. Notably, less than 25% of respondents endorsed ‘I did not think it was necessary’ and no one endorsed ‘I did not want to be stopped’.

### Group differences in reasons for not seeking NHS support

**Figure 1.**
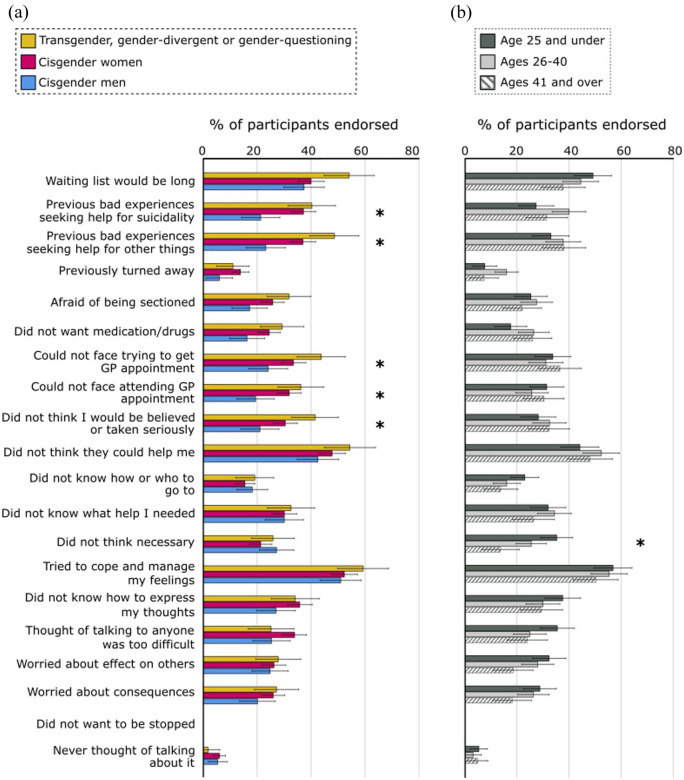
Effects of (a) gender and (b) age on reasons for not seeking help from the NHS. The horizontal axis displays the percentage of participants who endorsed each reason; group differences significant at an FDR-corrected threshold of p < 0.05 are marked with asterisks. Error bars reflect 95% confidence intervals.

A within-subjects main effect showed that participants rated the 20 reasons as differentially important in their decision to not seek NHS help (F [14.62, 10832.06] = 9.67, p < 0.001, partial η^2^ = 0.01); a main effect of Gender as a between-subject variable reflected different response patterns from autistic people of different genders (F [2, 741] = 9.28, p < 0.001, partial η^2^ = 0.02). Importantly, two-way interactions between Reasons and Gender (F [29.24, 10832.06] = 2.26, p < 0.001, partial η^2^ = 0.01), and between Reasons and Age (F [29.24, 10832.06] = 2.18, p < 0.001, partial η^2^ = 0.01), reflected that the magnitude of group differences differed across items.^1^ Significant differences between different Age and Gender groups, revealed by planned comparisons, are shown in Figure 1(a) and (b) (see Supplementary Table 2 for full details). As pertains to Gender, using cisgender men as the reference category, main effects reflected higher endorsement of previous bad experiences seeking help for suicidality, bad experiences seeking help for other things and feeling unable to face attending the GP in cisgender women and trans/gender-divergent participants. Trans/gender-divergent participants were also more likely than cisgender men to endorse feeling unable to face trying to make a GP appointment and feeling that they would not be taken seriously. A single highly significant effect of Age was seen for the reason ‘I did not think it was necessary’, which was endorsed significantly more frequently by the ⩽25 and 26–40 age groups than the ⩾41 age group.

**Figure 2.**
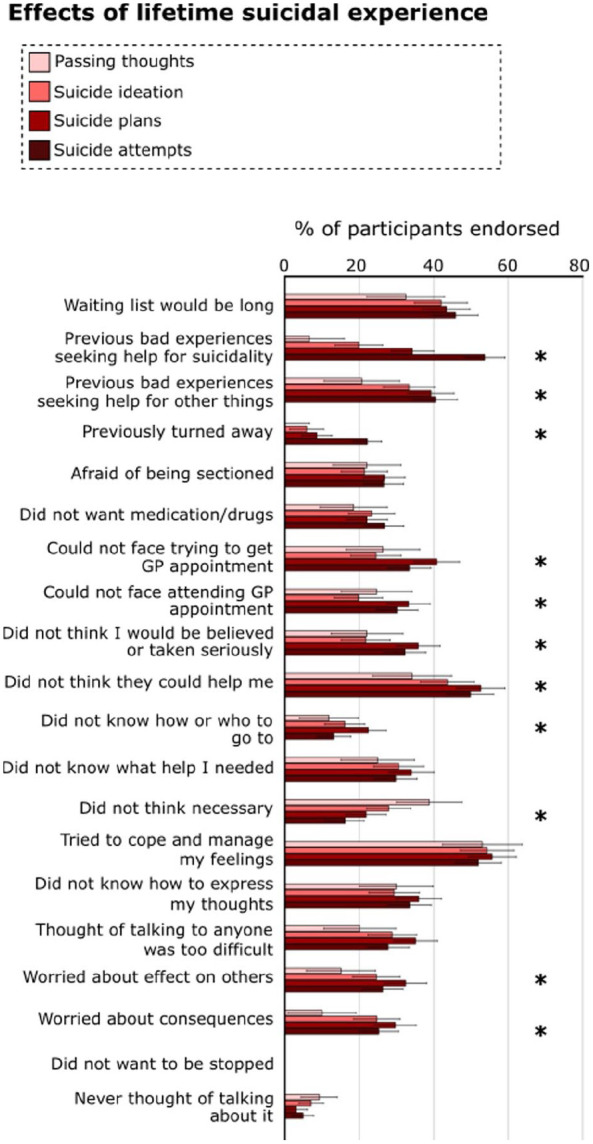
Effects of lifetime suicidal experience on reasons for not seeking help from the NHS. The horizontal axis displays the percentage of participants who endorsed each reason; group differences significant at an FDR-corrected threshold of p < 0.05 are marked with asterisks. Error bars reflect 95% confidence intervals.

Endorsement of reasons also differed significantly among individuals with differing lifetime experience of suicidality (Figure 2) (main effect of Lifetime suicidality: F [14.92, 744] = 7.77, p < 0.001, partial η^2^ = 0.03; interaction of Reasons and Lifetime suicidality: F [44.22, 10966.61] = 4.10, p < 0.001, partial η^2^ = 0.02).^2^ Here, main effects of Lifetime suicidality were reflected in greater endorsement of previous bad experiences, both in seeking help for suicidality and for other things, in individuals who had attempted suicide than in any other group; those who had attempted suicide were also significantly more likely to report having previously been turned away. Individuals with greater lifetime suicidality endorsed being unable to face making or attending a GP appointment, and feeling that they would not be believed, more than any other group. In contrast to individuals with passing thoughts of suicide, those who had attempted suicide were more likely to endorse believing that the NHS was unable to help, and more likely to endorse worrying about the effect on others and potential consequences for themselves. Those who had attempted suicide were also significantly less likely to endorse feeling that seeking help was unnecessary than those with passing thoughts and those with suicidal ideation without plans; in contrast to those who had made suicide plans, those who had attempted suicide were also less likely to express not knowing how or who to seek help from.

### Qualitative findings

**Figure 3.**
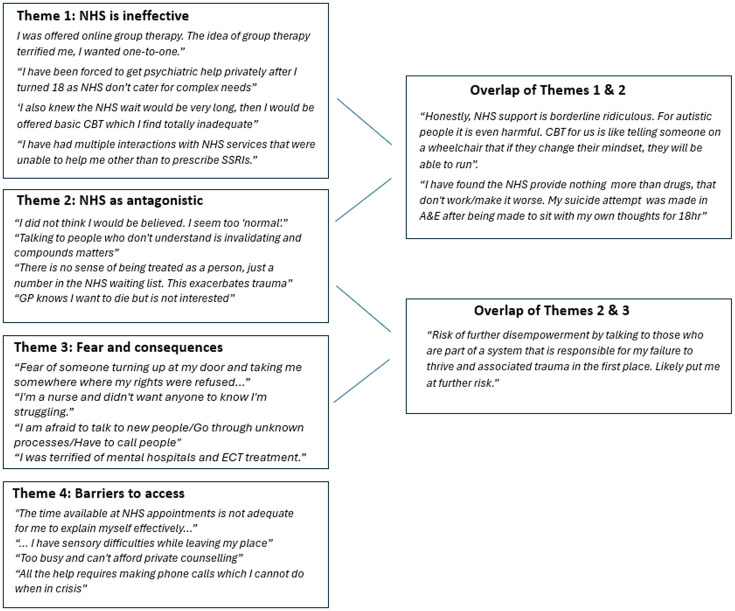
Thematic analysis and illustrative quotes.

Through thematic analysis of free-form responses, we interpreted four overlapping themes relating to reasons for not seeking NHS help (see Figure 3 for example quotations).

Theme 1 (‘NHS is ineffective’) includes subthemes that the specific support needed was unavailable through the NHS, the support offered by the NHS was unhelpful, and that the NHS was overburdened or lacked resources to help effectively. Being autistic was often related to these sentiments, with respondents expressing the NHS ‘does not understand autism’ and was unable to cater for ‘people like us’ with ‘complex needs’. Multiple respondents felt that the NHS was overly reliant on cognitive behavioural therapy (CBT) and antidepressant medication, which they believed were unsuitable for them, and that seeking NHS help would result in offer of the same support they had previously found unhelpful. Some respondents did not want to further burden what they perceived as an overstretched system, with one stating ‘they haven’t the money or time for my edge case’.

Theme 2 (‘NHS as antagonistic’) reflects subthemes where respondents did not seek NHS support due to experiences resulting in feelings of neglect, misunderstanding, invalidation or distrust. Several respondents expressed general distrust of medical professionals, while others shared that negative interactions with the NHS had exacerbated their poor mental health. Some respondents used expletives when describing their negative experiences, mocked the support offered by the NHS (‘Ha ha ha ha ha what help?!!!!!?’) or wrote in entirely uppercase letters, which we interpret as reflecting frustration and a sense of not being heard.

Theme 3 (‘Fear and consequences’) comprises subthemes related to potential repercussions of help-seeking, such as privacy breaches, stigma, unwanted treatment/sectioning and loss of control. Respondents were concerned about suicidality appearing on their medical records, with some believing breach of this information could negatively impact their job or family or be used against them in legal proceedings. Two respondents expressed concern that help-seeking could jeopardise their ability to seek gender-affirming healthcare in the future.

Theme 4 (‘Barriers to access’) includes subthemes regarding factors hindering respondents’ ability to seek help, such as lack of accommodations for autism offered by NHS clinics, being too distressed and/or incapacitated to seek support when experiencing suicidality, the need to involve parents/caregivers and a lack of time. Many responses related to communication challenges, particularly the need to make a phone call to access services.

Some responses included multiple reasons for not seeking NHS support and touched on multiple themes. For instance, prior experiences of unhelpful NHS support commonly overlapped with negative sentiments about the NHS (Themes 1 and 2). Themes 2 and 3 were also frequently linked, where distrust of the NHS related to understandable fear of approaching it. Access barriers (Theme 4) were sometimes linked to the NHS’s ‘bureaucracy and indifference’ (Theme 2) towards autistic people, with one respondent writing that any NHS help would require ‘pressing redial on [the] phone 20–70 times’.

## Discussion

With autistic people at increased risk of dying by suicide, understanding the factors that prevent potentially life-saving help-seeking behaviour is of utmost priority.

In our study of UK-based autistic adults, roughly 1 in 4 reported having sought NHS help when they last experienced suicidal thoughts or behaviours. NHS services – including mental health support such as therapy and counselling – are publicly funded and most UK residents are registered with a local NHS GP practice. However, our qualitative and quantitative analyses revealed a multitude of reasons why autistic people do not view the NHS as a source of support, including preference to cope independently, belief that the NHS cannot help (as it is ineffective or untrustworthy), fear of diverse consequences (unwanted treatment, effects on job or family, loss of control, etc.) and difficulty accessing support (communication challenges, etc.).

Notably, many reasons identified in our study have been previously reported as barriers autistic people face when accessing healthcare more broadly. An international survey of autistic adults found that not feeling understood, communication challenges related to making appointments by telephone or interacting with doctors and the waiting room environment were the most common barriers to accessing healthcare, with participants reporting that these barriers resulted in both their physical and mental health conditions going untreated (Doherty et al., 2022). Fear and distrust of NHS services has also emerged robustly in previous research as an impediment to help-seeking by autistic adults (Radev et al., 2024) and a factor that prevents autistic people from using tools designed to improve their healthcare experiences (Grant et al., 2024). It is essential to recognise, as clearly shown herein, that reticence towards help-seeking is a wholly appropriate response to ineffective treatment and physical and/or psychological harm from medical care (iatrogenic harm). Efforts to address the beliefs and feelings that prevent NHS-help seeking will be beneficial only so far as this and other public healthcare services are capable of safely and appropriately supporting autistic people presenting with suicidal thoughts. A systematic review and thematic meta-synthesis of autistic people’s experiences related to mental health support (Brede et al., 2022) concluded there was a need for ‘a more flexible, comprehensive and holistic approach’, which resonates with our participants’ complaints about the NHS’s limited range of mental health services, inability to support complex co-occurring conditions and inflexible communication methods. Our finding that no one endorsed the reason ‘I did not want to be stopped’ suggests respondents to our survey *do* desire support but have been let down by existing systems. Listening to autistic people and tailoring mental health services to address their needs is a crucial step for building public healthcare services capable of supporting autistic people and the trusting relationship necessary for engagement.

Our analyses also revealed differences in the most common reasons for not seeking NHS support for suicidality between sub-groups, indicating the need to make public healthcare services safe for minorities. Compared to cisgender men, cisgender women and trans/gender-divergent participants were more likely to endorse previous negative experiences with the NHS and not being able to face a GP appointment. Trans/gender-divergent participants were also more likely to endorse that they would not be believed or taken seriously by the NHS. These findings correspond with previous reports of additional barriers to healthcare faced by autistic women and gender minorities (Grove et al., 2023; Koffer Miller et al., 2022), including recent evidence that trans/gender-divergent autistic people have more negative healthcare experiences in general than their cisgender autistic and trans/gender-divergent non-autistic counterparts (Green et al., 2025). While it has been previously reported that women at risk of suicide are more likely than men to seek GP support (Mok et al., 2021), if autistic women or LGBTQIA+ individuals have more negative healthcare experiences, this could inhibit future help-seeking when it is direly needed. Previous studies of LGBTQIA+ communities have flagged discrimination and lack of understanding as important barriers to mental health support (Crockett et al., 2022; McNair & Bush, 2016; Silveri et al., 2022), and our findings suggest this extends to autistic LGBTQIA+ individuals. We also note the large proportion (21%) of trans/gender-divergent participants in our study, which reflects the overlap between being autistic/neurodivergent and being trans/gender-divergent (Warrier et al., 2020), higher suicidality in trans/gender-divergent individuals (Erlangsen et al., 2023) and particularly heightened suicidality in individuals who are autistic *and* trans/gender-divergent (Mournet et al., 2024).

Further group comparisons also revealed that, compared to older participants (age ⩾ 41), younger participants were more likely to believe NHS support was unnecessary when experiencing suicidality. With striking increases in the incidence of common mental health conditions in young adults in the United Kingdom in recent decades (Dykxhoorn et al., 2024), younger respondents may have more experience self-managing mental health challenges and thus be less likely to medicalise suicidality. Although our results did not show significant age-related differences for endorsement of the reason ‘I tried to cope and manage my feelings’, a study of help-seeking among young people with a history of self-injury found a preference for online versus in person support, such as a GP (Frost & Casey, 2016). Younger autistic people may be more likely to use forms of support other than the NHS to cope with suicidality, such as online communities or mental health apps; future research could explore this possibility.

Striking differences in reasons for not seeking NHS support also emerged between sub-groups with different levels of lifetime suicidality. Participants who had attempted suicide were more likely to endorse previous bad experiences seeking help for suicidality/other things, previously being turned away/rejected, believing they could not be helped and being worried about consequences as reasons for not seeking NHS support, but were less likely to endorse that help was unnecessary. Given that history of suicide attempts is one of the strongest predictors of future attempts and death in general population samples (Bostwick et al., 2016), if this effect extends to autistic people, improving the quality and accessibility of NHS services for autistic people at especially high risk is essential for building trust and encouraging help-seeking behaviours.

A policy brief arising from an international meeting of autism researchers and stakeholders identifies understanding and removing barriers to mental health support as the top community priority for suicide prevention (Cassidy, Goodwin, et al., 2021). Our findings underscore the presence and prevalence of such barriers, stressing the urgent need to tailor NHS services to meet the unique experiences and requirements of autistic people. Addressing barriers to help-seeking for suicidality requires systemic changes that prioritise trust-building, accessibility and inclusivity, as well as development of efficacious and acceptable ways of supporting autistic people experiencing suicidal feelings. Autistic individuals often encounter stigma, miscommunication and a lack of understanding within healthcare systems, which contribute to distrust and disengagement (Camm-Crosbie et al., 2019; Crane et al., 2019; Grant et al., 2024; Radev et al., 2024). As clinicians report greater self-efficacy screening for suicide risk among non-autistic people (Cervantes et al., 2023, 2024, 2025; Jager-Hyman et al., 2020), a clear place to start is training healthcare professionals in autism awareness and adapting communication approaches. With telephone calls inaccessible for many autistic people (Howard & Sedgewick, 2021), online appointment booking systems could greatly facilitate help-seeking for mental health. By extending beyond the traditional healthcare system, such as tele-health appointments or self-guided digital health tools, suicide prevention efforts can become more inclusive and effective (Torok et al., 2020). Transparency about the next steps after someone discloses suicidality to their GP, expressed clearly through a pamphlet or website, could help mitigate fear of consequences or loss of control. As a step towards rebuilding trust, any and all potential approaches should be considered and designed collaboratively with autistic people as equal partners.

### Limitations and future directions

While the present study offers practical contributions towards efforts to facilitate help-seeking when autistic people experience suicidality, there are several notable limitations. Our findings are culture-bound, though they may generalise to other countries with public healthcare systems. Like many online surveys of autistic people (Rødgaard et al., 2022), our self-selecting sample was biased towards cisgender women and highly educated participants and is thus unrepresentative of the autistic population as a whole – especially those with learning disabilities and ethnic minorities who face additional intersectional challenges related to healthcare (Lindsay et al., 2024). Given our focus on reasons for *not* seeking help, our data only reflects participants willing to share their experiences with suicide and ideas for suicide prevention: it is probable that many individuals who do not seek support for suicidality refrain from participating in research, as has been reported for non-autistic cisgender men (Choi et al., 2017). As such, some reasons for not seeking NHS help might be un- or under-represented here, warranting further research involving more diverse groups. Similarly, additional research is needed to understand autistic people’s experiences seeking support for suicidality from more diverse sources, such as autism-specific services, help lines or peer support programmes.

In this quantitative approach, we were unable to contextualise findings with several important pieces of information, such as the recency of participants’ last suicidal thoughts, last approach to NHS services or nature of previous healthcare encounters, including those related to autism assessment. These details may have had notable impacts on the experiences participants described and should be more comprehensively explored in future research. Having asked broadly why participants did not seek help ‘from the NHS’, the majority of our findings, such as negative previous encounters, cannot be localised to specific services or professionals within this extensive system. Where previous studies have examined autistic people’s perceptions of clinical risk assessment and treatment for suicidality in specific services (Cervantes et al., 2024), future research should aspire to greater specificity and deeper insights into negative experiences when seeking help for suicidality using participatory approaches.

## Conclusion

With clinicians commonly reporting limited knowledge and low confidence working with autistic people (Maddox et al., 2020), a clear place to start with adjustments to services to address suicide risk is by listening to the autistic community to obtain specific recommendations and ensure services are both respectful and responsive to their needs. Our study identified numerous barriers to seeking NHS help for suicidality, including preference for self-management, belief that the NHS is ineffective and overstretched, distrust and fear of consequences. Future studies should aim to understand barriers to help-seeking for suicidality among more diverse groups of autistic people in various countries and to gain deeper insights into experiences with specific services. Ultimately, building healthcare systems that provide appropriate help for autistic people and that autistic people perceive as trustworthy and effective will lead to better well-being and fewer lives lost to suicide.

## Supplemental Material

sj-docx-1-aut-10.1177_13623613251370789 – Supplemental material for ‘I did not think they could help me’: Autistic adults’ reasons for not seeking public healthcare when they last experienced suicidality Supplemental material, sj-docx-1-aut-10.1177_13623613251370789 for ‘I did not think they could help me’: Autistic adults’ reasons for not seeking public healthcare when they last experienced suicidality by Tanya L Procyshyn, Rachel L Moseley, Sarah J Marsden, Carrie Allison, Tracey Parsons, Sarah Cassidy, Mirabel Pelton, Elizabeth Weir, Tanatswa Chikaura, Holly Hodges, David Mosse, Ian Hall, Lewis Owens, Jon Cheyette, David Crichton, Jacqui Rodgers and Simon Baron-Cohen in Autism

## Notes

1. Although not the focus of our analysis, several covariates displayed significant interactions with ‘Reasons’: Diagnostic status (F [14.62, 10832.06] = 3.28, p < 0.001, partial η^2^ = 0.004); Educational attainment (F [14.62, 10832.06] = 1.97, p = 0.015, partial η^2^ = 0.003); and Current employment (F [14.62, 10832.06] = 1.94, p = 0.017, partial η^2^ = 0.003). These covariates did not exert main effects on responses.

2. Main effects of Gender (F [1, 744] = 16.40, p < 0.001, partial η^2^ = 0.02) and Age (F [1, 744] = 6.63, p = 0.01, partial η^2^ = 0.01) persisted, as did interactions between Gender and Reasons (F [14.74, 10966.61] = 2.52, p = 0.001, partial η^2^ = 0.003), and Age and Reasons (F [14.74, 10966.61] = 3.93, p < 0.001, partial η^2^ = 0.005). There were also interactions between Reasons and Diagnostic status (F [14.74, 10966.61] = 2.71, p < 0.001, partial η^2^ = 0.004), and as previously, a within-subjects effect of Reasons (F [14.74, 10966.61] = 10.51, p < 0.001, partial η^2^ = 0.01).
