# Supplementary material for: ‘I did not think they could help me’: Autistic adults’ reasons for not seeking public healthcare when they last experienced suicidality
Source: Autism. 2025 Sep 15;29(11):2677–90. doi: 10.1177/13623613251370789 (PMC12531389; doi:10.1177/13623613251370789)
Supplement: sj-docx-1-aut-10.1177_13623613251370789 – Supplemental material for ‘I did not think they could help me’: Autistic adults’ reasons for not seeking public healthcare when they last experienced suicidality [file sj-docx-1-aut-10.1177_13623613251370789.docx]

**Supplementary Materials**

**Methods**

***Dataset and participant selection***

This study focused on a subset of participants recruited as part of a larger project on priorities for suicide prevention in relation to autism run from January to July 2024. This project was advertised on Facebook, Youtube, Twitter/X, Instagram, and the website of the charity who initiated the study and funded its promotion (redacted). To be eligible, participants had to be 16 or over and autistic, whether formally-diagnosed or self-identifying. The study was open to international participants, though promotion centered on the UK population. A group of over 90 individuals (autistic people and their family members, including those bereaved by the suicide of an autistic person) provided guidance on communication and advertisement of the survey.

The total number of datasets received as part of the overall project was 1,525. The breakdown of discarded datasets (n=473) was as follows: respondents who were not autistic (n=41); those who had never experienced suicidal thoughts, who therefore did not respond to questions necessary for analyses (n=23); duplicates or abusive responses (n=18); and responses deemed likely to be bots (n=5; 16 other ‘potential’ bots were deemed genuine and retained). For the purpose of the present analyses, we also discarded datasets from participants who resided outside of the UK and/or who did not reach or complete the pertinent questions for this analysis (n=386); regrettably, this also included 208 participants who completed the pilot survey.

**Pilot survey:** To ensure acceptability of the survey, a 2-week pilot period was conducted. The pilot survey included a list of 15 reasons for not seeking NHS support and was completed by 208 participants. Based on data from the pilot survey, the following five reasons were added: ‘I was afraid of being sectioned’; ‘I did not want to be prescribed or forced to take medication/drugs’; ‘I could not face trying to get a GP appointment’; ‘I could not face attending a GP appointment’; and ‘I did not think I would be believed or taken seriously’.

**Supplementary Table 1.** Binary logistic regression exploring demographic factors predicting respondents who provided optional free-form responses (n = 754).

| **Predictor** | **Estimate** | **SE** | **Z** | **p** | **Odds ratio** |
| --- | --- | --- | --- | --- | --- |
| Intercept | -1.41412 | 0.405 | -3.4916 | < .001 | 0.243 |
| *Ethnicity* |  |  |  |  |  |
| 0 – 1 | -0.48843 | 0.277 | -1.7656 | 0.077 | 0.614 |
| *AutismStatus:* |  |  |  |  |  |
| 0 – 1 | 0.19245 | 0.192 | 1.0044 | 0.315 | 1.212 |
| *Age group:* |  |  |  |  |  |
| 2 – 1 | 0.54014 | 0.253 | 2.1334 | 0.033* | 1.716 |
| 3 – 1 | 0.66483 | 0.252 | 2.6362 | 0.008** | 1.944 |
| *Gender:* |  |  |  |  |  |
| 2 – 1 | 0.00366 | 0.255 | 0.0143 | 0.989 | 1.004 |
| 3 – 1 | -0.30055 | 0.302 | -0.995 | 0.32 | 0.74 |
| *Education:* |  |  |  |  |  |
| 0 – 1 | 0.01353 | 0.199 | 0.0681 | 0.946 | 1.014 |
| *Employment3:* |  |  |  |  |  |
| 0 – 1 | -0.08746 | 0.222 | -0.3934 | 0.694 | 0.916 |

**Supplementary Table 2**

Planned comparisons: main effects of gender, age and lifetime suicidality on reasons for not seeking NHS help.

| **Reasons for not seeking NHS help** | **Effects of gender** | **Effects of age** | **Effects of lifetime suicidal experience** |
| --- | --- | --- | --- |
| Waiting list would be long | F (2, 741) = 4.34, p = .013 | F (2, 741) = 2.01, p = .135 | F (3, 744) = 1.59, p = .190. |
| Previous bad experiences seeking help for suicidality | F (2, 741) = 8.00, p <.001, partial η^2^ = .02.  Cisgender men [M: .24, SD: .43] vs.:   - Transgender, gender-divergent and questioning participants [M: .42, SD: .49]: mean contrast = .19, p = .001. - Cisgender women [M: .37, SD: .48]: mean contrast = .16, p < .001. | F (2, 741) = 3.62, p = .027 | F (3, 744) = 33.39, p < .001, partial η^2^ = .12.  Suicide attempt group [M: .56, SD: .48] vs.:   - Passing thoughts [M: .06, SD: .24]: mean contrast = .48, p < .001. - Suicide ideation [M: .19, SD: .39]: mean contrast = .34, p < .001. - Suicide plans [M: .33, SD: .47]: mean contrast = .19, p < .001. |
| Previous bad experiences seeking help for other things | F (2, 741) = 9.62, p <.001, partial η^2^ = .03.  Cisgender men [M: .25, SD: .44] vs.:   - Transgender, gender-divergent and questioning participants [M: .48, SD: .50]: mean contrast = .25, p < .001. - Cisgender women [M: .37, SD: .48]: mean contrast = .14, p = .002. | F (2, 741) = .58, p = .559 | F (3, 744) = 4.18, p = .006, partial η^2^ = .02.  Suicide attempt group [M: .42, SD: .49] vs.:   - Passing thoughts [M: .20, SD: .40]: mean contrast = .20, p = .001. - Suicide ideation [M: .33, SD: .47]: mean contrast = .07, p = .129. - Suicide plans [M: .39, SD: .49]: mean contrast = .01, p = .789. |
| Previously turned away or referral rejected | F (2, 741) = 3.47, p = .032. | F (2, 741) = 4.33, p = .014 | F (3, 744 = 15.78, p < .001, partial η^2^ = .06.  Suicide attempt group [M: .23, SD: .42] vs.:   - Passing thoughts [M: 0, SD: 0]: mean contrast = .22, p < .001. - Suicide ideation [M: .05, SD: .23]: mean contrast = .16, p < .001. - Suicide plans [M: .08, SD: .28]: mean contrast = .14, p < .001. |
| Afraid of being sectioned | F (2, 741) = 4.05, p = .018. | F (2, 741) = .65, p = .520. | F (3, 744) = .78, p = .503. |
| Did not want medication/drugs | F (2, 741) = 3.45, p = .032. | F (2, 741) = 2.37, p = .095. | F (3, 744) = .41, p = .419. |
| Could not face trying to get a GP appointment | F (2, 741) = 5.75, p = .003, partial η^2^ = .02.  Cisgender men [M: .25, SD: .43] vs.:   - Transgender, gender-divergent and questioning participants [M: .41, SD: .49] vs. mean contrast = .20, p < .001. - Cisgender women [M: .33, SD: .47]: mean contrast = .09, p = .032. | F (2, 741) = .53, p = .591. | F (3, 744) = 4.74, p = .003, partial η^2^ = .02.  Suicide attempt group [M: .34, SD: .47] vs.:   - Passing thoughts [M: .26, SD: .44]: mean contrast = .07, p = .230. - Suicide ideation [M: .25, SD: .43]: mean contrast = .09, p = .051. - Suicide plans [M: .41, SD: .49]: mean contrast = .07, p = .087. |
| Could not face attending a GP appointment | F (2, 741) = 5.94, p = .003, partial η^2^ = .02.  Cisgender men [M: .17, SD: .38] vs.:   - Transgender, gender-divergent and questioning participants [M: .32, SD: .47]: mean contrast = .17, p = .003. - Cisgender women [M: .31, SD: .47]: mean contrast = .13, p = .003. | F (2, 741) = .86, p = .425. | F (3, 744) = 3.47, p = .016, partial η^2^ = .01.  Suicide attempt group [M: .31, SD: .46] vs.:   - Passing thoughts [M: .24, SD: .43]: mean contrast = .06, p = .331. - Suicide ideation [M: .20, SD: .40]: mean contrast = .10, p = .018. - Suicide plans [M: .33, SD: .47]: mean contrast = .03, p = .461. |
| Did not think I would be believed or taken seriously | F (2, 741) = 6.57, p = .001, partial η^2^ = .02.  Cisgender men [M: .22, SD: .42] vs.:   - Transgender, gender-divergent and questioning [M: .39, SD: .49] vs. mean contrast = .21, p < .001. - Cisgender women [M: .30, SD: .46]: mean contrast = .09, p = .028. | F (2, 741) = .48, p = .619. | F (3, 744) = 4.32, p = .005, partial η^2^ = .02.  Suicide attempt group [M: .33, SD: .47] vs.:   - Passing thoughts [M: .21, SD: .41]: mean contrast = .10, p = .074. - Suicide ideation [M: .22, SD: .41]: mean contrast = .11, p = .018. - Suicide plans [M: .35, SD: .48]: mean contrast = .04, p = .401. |
| Did not think they could help me | F (2, 741) = 1.82, p = .163. | F (2, 741) = 1.26, p = .284. | F (3, 744) = 3.36, p = .018, partial η^2^ = .01.  Suicide attempt group [M: .51, SD: .50] vs.:   - Passing thoughts [M: .34, SD: .48]: mean contrast = .16, p = .013. - Suicide ideation [M: .43, SD: .50]: mean contrast = .06, p = .203. - Suicide plans [M: .52, SD: .50]: mean contrast = .04, p = .574. |
| Did not know how or who to go to | F (2, 741) = .57, p = .564. | F (2, 741) = 2.62, p = .073. | F (3, 744) = 3.06, p = .028, partial η^2^ = .01.  Suicide attempt group [M: .14, SD: .35] vs.:   - Passing thoughts [M: .11, SD: .31]: mean contrast = .01, p = .781. - Suicide ideation [M: .16, SD: .37]: mean contrast = .03, p = .414. - Suicide plans [M: .22, SD: .42]: mean contrast = .10, p = .007. |
| Did not know what help I needed | F (2, 741) = .13, p = .881. | F (2, 741) = 1.19, p = .304. | F (3, 744) = .86, p = .463. |
| Did not think necessary | F (2, 741) = 1.35, p = .260. | F (2, 741) = 9.81, p < .001, partial η^2^ = .03.  41 and above age group [M: .14, SD: .35] vs.:   - 25 and younger [M: .33, SD: .47]: mean contrast = .22, p < .001. - 26-40 years [M: .25, SD: .43]: mean contrast = .12, p = .012. | F (3, 744) = 7.19, p < .001, partial η^2^ = .03.  Suicide attempt group [M: .17, SD: .38] vs.:   - Passing thoughts [M: .39, SD: .49]: mean contrast = .23, p < .001. - Suicide ideation [M: .28, SD: .45]: mean contrast = .12, p = .004. - Suicide plans [M: .21, SD: .41]: mean contrast = .06, p = .140. |
| Tried to cope and manage my feelings | F (2, 741) = 1.02, p = .360. | F (2, 741) = .69, p = .502. | F (3, 744) = .23, p = .878. |
| Did not know how to express my thoughts | F (2, 741) = 2.06, p = .128. | F (2, 741) = 1.59, p = .205. | F (3, 744) = .81, p = .486. |
| Thought of talking to anyone was too difficult | F (2, 709) = 2.96, p = .052. | F (2, 709) = 3.36, p = .035. | F (3, 744) = 2.53, p = .056. |
| Worried about effect on others | F (2, 741) = .15, p = .858. | F (2, 741) = 3.65, p = .027. | F (3, 744) = 3.43, p = .017, partial η^2^ = .01.  Suicide attempt group [M: .28, SD: .45] vs.:   - Passing thoughts [M: .14, SD: .35]: mean contrast = .11, p = .043. - Suicide ideation [M: .25, SD: .43]: mean contrast = .02, p = .693. - Suicide plans [M: .32, SD: .47]: mean contrast = .06, p = .130. |
| Worried about consequences | F (2, 741) = 1.26, p = .285. | F (2, 741) = 2.35, p = .096. | F (3, 744) = 4.36, p = .005, partial η^2^ = .02.  Suicide attempt group [M: .26, SD: .44] vs.:   - Passing thoughts [M: .09, SD: .29]: mean contrast = .15, p = .005. - Suicide ideation [M: .25, SD: .43]: mean contrast = .005, p = .903. - Suicide plans [M: .29, SD: .44]: mean contrast = .04, p = .266. |
| Did not want to be stopped | Endorsed by no participants. | Endorsed by no participants. | Endorsed by no participants. |
| Never thought of talking about it | F (2, 741) = 1.46, p = .234. | F (2, 741) = .50, p = .607. | F (3, 744) = 1.96, p = .119. |

*Note*. Differences significant at an FDR-corrected threshold of p < .05 are displayed in black.
